# Pituitary Adenylate Cyclase-Activating Polypeptide Reverses Ammonium Metavanadate-Induced Airway Hyperresponsiveness in Rats

**DOI:** 10.1155/2015/787561

**Published:** 2015-06-14

**Authors:** Mounira Tlili, Sonia Rouatbi, Badreddine Sriha, Khémais Ben Rhouma, Mohsen Sakly, David Vaudry, Olivier Wurtz, Olfa Tebourbi

**Affiliations:** ^1^Laboratory of Integrated Physiology, Science Faculty of Bizerte, Carthage University, 7021 Zarzouna, Tunisia; ^2^National Institute of Health and Medical Research (INSERM), U982, 76821 Mont-Saint-Aignan Cedex, France; ^3^Institute for Research and Innovation in Biomedicine (IRIB), Normandy University, 76821 Mont-Saint-Aignan Cedex, France; ^4^Laboratory of Neuronal and Neuroendocrine Differentiation and Communication, Rouen University, 76821 Mont-Saint-Aignan Cedex, France; ^5^Laboratory of Physiology and Functional Exploration, CHU Farhat Hached, 4000 Sousse, Tunisia; ^6^Laboratory of Pathologic Anatomy, CHU Farhat Hached, 4000 Sousse, Tunisia

## Abstract

The rate of atmospheric vanadium is constantly increasing due to fossil fuel combustion. This environmental pollution favours vanadium exposure in particular to its vanadate form, causing occupational bronchial asthma and bronchitis. Based on the well admitted bronchodilator properties of the pituitary adenylate cyclase-activating polypeptide (PACAP), we investigated the ability of this neuropeptide to reverse the vanadate-induced airway hyperresponsiveness in rats. Exposure to ammonium metavanadate aerosols (5 mg/m^3^/h) for 15 minutes induced 4 hours later an array of pathophysiological events, including increase of bronchial resistance and histological alterations, activation of proinflammatory alveolar macrophages, and increased oxidative stress status. Powerfully, PACAP inhalation (0.1 mM) for 10 minutes alleviated many of these deleterious effects as demonstrated by a decrease of bronchial resistance and histological restoration. PACAP reduced the level of expression of mRNA encoding inflammatory chemokines (MIP-1*α*, MIP-2, and KC) and cytokines (IL-1*α* and TNF-*α*) in alveolar macrophages and improved the antioxidant status. PACAP reverses the vanadate-induced airway hyperresponsiveness not only through its bronchodilator activity but also by counteracting the proinflammatory and prooxidative effects of the metal. Then, the development of stable analogs of PACAP could represent a promising therapeutic alternative for the treatment of inflammatory respiratory disorders.

## 1. Introduction

Vanadium is a naturally occurring ubiquitous transition metal present in most plant and animal tissues [1]. This trace element is distributed extensively in nature and used increasingly in various heavy industries, such as steel and oil. It is mainly used in the production of nonferrous alloys, most resistant carbon steel, as well as in chemical, glass, paint, varnish, ceramic, and photographic industries [2]. Vanadium is a major trace metal in fossil fuels, and combustion of these materials is responsible for environmental pollution with this element.

Both acute and chronic poisonings have been described in workers engaged in the industrial production and use of vanadium, indicating that vanadium exposures are associated with upper and lower respiratory irritative symptoms and cause airway hyperresponsiveness and inflammation [3]. Concurrent changes in pulmonary function, such as decreased forced vital capacity and forced expiratory volume, signs of respiratory distress, impaired lung function, increased pulmonary reactivity, and histological alterations are reported in vanadium-exposed laboratory animals [4]. It is suggested from clinical and animal studies that the pulmonary effects of vanadate involve a direct action on the airway smooth muscle. Bronchoconstriction induced by vanadate can be ascribed to an increased level of cytoplasmic calcium (Ca^2+^_cyt_) consecutive to Ca^2+^ release from intracellular stores due to the combined production of inositol phosphate and inhibition of Ca^2+^-ATPase [4, 5]. In parallel to its direct action on airway smooth muscles, the vanadate exposure affects the local immune compartment as illustrated by alterations of immune cell phagocytic activity, lymphoproliferative responsiveness, increased inflammatory cell infiltration in pulmonary tissue [6], and upregulated expression of a variety of inflammatory cytokines and their receptors [7]. Furthermore, many studies showed vanadium-mediated generation of reactive oxygen species (ROS) participating in its toxicity. In biological systems, pentavalent vanadium is converted to a tetravalent form via a glutathione-dependent process. During this reduction process, molecular oxygen is reduced to superoxide anion (^∙^O_2_^−^) and then to hydrogen peroxide (H_2_O_2_) via dismutation [8]. Hence, ROS may potentiate lung conditions such as bronchitis, asthma, bronchopneumonia, lung fibrosis, and pulmonary inflammation.

Pituitary adenylate cyclase-activating polypeptide (PACAP) is a 38-amino acid peptide that was first isolated from an ovine hypothalamic extract on the basis of its ability to stimulate cAMP formation in anterior pituitary cells [9]. Among numerous properties, PACAP appears to be a potent dilatator of human bronchi. Produced in human pulmonary tract, it is also involved in the endogenous regulation of the airway tone [10, 11]. In particular, PACAP inhibits smooth muscle tone induced by acetylcholine, histamine or methacholine in guinea-pig trachea [12] and suppresses the increase in airway hyperresponsiveness induced by ozone exposure [13]. Furthermore, PACAP exhibits modulatory activities on innate immune system. PACAP inhibits the release of both IL-6 and IL-12 as well as TNF-*α* from lipopolysaccharide-stimulated macrophages [14–18] and NO production in a dose- and time-dependent manner [19]. Based on these observations, in the present study we hypothesized that PACAP may reverse the adverse effects of vanadium on lungs and assessed the ability of PACAP to decrease bronchoconstriction and tissue damages induced by an acute ammonium vanadate (AMV) exposure in rats.

## 2. Materials and Methods

### 2.1. Animals

Male Wistar rats (200–250 g) were housed under controlled temperature, in twelve-hour day/night cycle with food and water available* ad libitum*. Animals were cared for in compliance with the code of practice for the Care and Use of Animals for Scientific Purposes. Approval for these experiments was obtained from the Medical Ethical Committee for the Care and Use of Laboratory Animals of Pasteur Institute of Tunis (Approval number LNFP/Pro 152012).

### 2.2. Chemicals

Ammonium metavanadate NH_4_VO_3_ was purchased from Sigma (St. Louis, MO, USA). The 38-amino acid form of PACAP was generously provided by Professor A. Fournier.

### 2.3. Aerosols Generation and Treatments

Rats were randomly divided into three experimental groups (8 animals/group). According to the groups, experimental animals were exposed to saline aerosols only (control rats), to ammonium metavanadate aerosols (AMV; 5 mg/m^3^/h) for 15 minutes followed by a 10-minute exposure to saline aerosol (AMV-treated animals) or to AMV aerosols (5 mg/m^3^/h) for 15 minutes followed by a 10-minute exposure to PACAP-containing aerosols (0.1 mM, AMV + P_38_ treated group). Aerosols were generated through a DeVibliss nebulizer (Ref. 123016 Marquette Medical products, Englewood Co., USA) connected to a compressor (flow rate 100 mL/s) with a flow rate of 0.1 mL/s in a rigid plastic chamber placed over the rat body.

### 2.4. Lung Resistance Measurement

Animals were anesthetized with ketamine (100 mg/Kg). The necks were opened; the trachea was exposed by a midline incision and a tracheal cannula was inserted. A second balloon tipped catheter was inserted into the lower third of the oesophagus and connected to a pressure transducer to measure the intraoesophageal pressure. A pneumotachograph (PTG; 8431B, Hnans Rudolph, Kansas, USA) was connected to the tracheal cannula at the time of measurement of the flow rate whose period was set at 10 seconds to avoid change in ventilation due to the PTG dead volume. The PTG was connected to a differential pressure transducer. Both transducers were assembled together with connecting valves to ease the calibration and clearing of the oesophageal catheter (Pneumomultitest ERMS, Toulouse, France). The total lung resistance (*L*_*R*_) was measured 5 minutes after each aerosol. To determine the optimal *L*_*R*_ response, increasing doses of AMV were administrated for 15 minutes. Total lung resistance (*L*_*R*_) was determined using a first-order mechanical model of the lung.

### 2.5. Determination of Vanadium Lung Contents

Total vanadium was determined in rat lungs using inductively coupled plasma atomic emission spectrometry (ICP-AES) [20]. The standard solution of vanadium used in this assay is obtained after the dissolution of NH_4_VO_3_ in distilled water. Fractions of tissues were lyophilized, weighed, and digested in 2 mL of concentrated HNO_3_ in pressurized Teflon containers at 160°C for 3 h. After cooling at room temperature, samples were diluted with 10 mL of deionized water. Vanadium concentration was calculated in *μ*g/g of the dry mass of tissues.

### 2.6. Biochemical Analyses

Four hours after treatment, experimental rats were euthanatized and the lungs were recovered, weighed, and homogenized in phosphate buffer saline pH 7.4 using an Ultra-Turrax homogenizer. The supernatant obtained after centrifugation at 10,000 g (10 min) was used for biochemical analyses. Protein content was determined by a spectrophotometric Hartree method [21]. The generation of malondialdehyde (MDA) was used as a marker for lipid peroxidation, which was estimated by the method of Draper and Hadley [22]. Reduced glutathione (GSH) and oxidized glutathione (GSSG) were measured as described by Tietze [23]. The reducing power (GSH/GSSG ratio) was also calculated. Lung homogenates were used for endogenous antioxidant enzyme activities. Catalase (CAT) activity was measured at 25°C according to the method of Aebi [24]. Glutathione peroxidase (GPx) activity was measured according to Nakamura et al. [25]. Measurement of the total superoxide dismutase (SOD) activity was performed according to Misra and Fridovich [26] based on the inhibition of autoxidation of epinephrine. Characterization of SOD isoforms was performed using KCN (3 mmol·L^−1^) as a Cu-Zn inhibitor or H_2_O_2_ (3 mmol·L^−1^), which affects CuZn-SOD, whereas Mn-SOD is insensitive to both inhibitors [26]. Nitric oxide (NO) levels were determined by quantification of NO metabolites, nitrite, and nitrate, according to the previously described method of Green et al. [27]. Lung oxygen peroxide (H_2_O_2_) was measured using commercial kits from Biomaghreb (Tunisia) according to Kakinuma et al. [28].

### 2.7. Histological Analyses

Immediately after sacrifice, lungs were harvested, washed with ice cold saline, and fixed overnight at room temperature in paraformaldehyde 4% in 0.1 M phosphate buffer, pH 7.4. The samples were dehydrated with ethanol and toluene series and embedded in paraffin. Serial sections (5 *μ*m) were mounted on gelatin-coated glass slides, cut, and stained with hematoxylin and eosin (H&E) for histopathological analysis, using the trichrome Masson's technique for the morphometric study. The quantitative measurements were made with a computerized image analysis system using Image-Pro Plus version 4.5 software (Media Cybernetics Inc., Silver Spring, MD, USA). The protocol was systematized into three phases: capture, processing, and quantification. The images were captured at a size of 551/400 pixels at ×40, ×100, and ×200 magnifications. All the bronchi found to be smaller than or equal to the size of the histological field were captured. In order to only study the bronchi cut perpendicularly to their longitudinal axis, all the bronchi whose greatest diameter was at least twice as large as the least diameter were discarded. Airway size defined by the reticular basement membrane perimeter (pbm), the total area of the airway smooth muscle layer (ASM_area_), and the internal bronchial lumen diameter were measured by planimetry according to the method of James et al. [29]. The main outcome of interest was the ASM thickness (ASMT) which is the ASM_area_ normalized for airway size by dividing by the pbm (ASM_area_/pbm) and expressed in *μ*m. Thereafter, the airways were divided into three categories according to their pbm, as described by Sapienza et al. [30]: pbm ≤ 1 mm (small), pbm > 1 mm and ≤2 mm (medium), and pbm > 2 mm (large). The measurements were made using 10 randomly chosen medium-sized bronchi per animal (*n* = 8 for each experimental group).

### 2.8. Bronchoalveolar Lavages and Alveolar Macrophages (AM) Isolation

Four hours after treatment, rats were anesthetized with ketamine (100 mg/Kg). Lungs were washed* in situ* with 6 mL of warm phosphate-buffered saline (PBS) in the presence of EDTA (0.6 mM). This procedure was repeated 8 times. The bronchoalveolar lavage fluid (BALF) was immediately centrifuged at 1500 rpm for 5 minutes. The harvested cells were immediately processed for total RNA extraction for subsequent quantitative PCR experiments or cultured in RPMI1640 culture medium supplemented with 5% foetal bovine serum (FBS) at 37°C and 5% CO_2_ for 24 hours. To determine the adherent and nonadherent cell numbers, the culture medium was removed and the nonadherent cells in suspension were counted while the remaining adherent cells were counted after trypsin-EDTA treatment.

### 2.9. RNA Extraction and Quantitative Real Time-PCR (Q-PCR) Analysis

Total RNAs were extracted from BALF's cells using Tri-reagent (Invitrogen) and Nucleospin RNA II kit (Macherey-Nagel) according to the manufacturer's instructions. From each sample, 1.5 *μ*g of total RNA was converted into single stranded cDNA using the ImPromII reverse transcriptase kit (Promega) with random primers (0.5 *μ*g/mL). Real time-PCR experiments were performed and monitored by ABI Prism 7500 Sequence Detection System (Life Technologies) in the presence of a 1x Mastermix (Applied Biosystems) containing preset concentrations of dNTPs, MgCl_2_, SYBR green reporter dye, and specific primer pairs. Rat glyceraldehydes-3-phosphate dehydrogenase (GAPDH) cDNA was used as control. Results were calculated using the 2^−ΔCt^ method, where ΔCt is the difference in the Ct values for the target gene and the reference gene. Primers used were as follows: IL-1*α* forward 5′AAGACAAGCCTGTGTTGCTGAAGG and reverse 5′TCCCAGAAGAAAATGAGGTCGGTC; TNF-*α* forward 5′AAGGCTGCCCCGACTATGTGC and reverse 5′TGGCGGAGAGGAGGCTGACTT; IL-6 Forward 5′GCCTTCTTGGGACTGATGTTGTTG and reverse 5′TGGTATCCTCTGTGAAGTCTCCTCTCC; MIP-1*α* forward 5′CCAGCAGCCTTTGGTCCCAG and reverse 5′CAGGTCTCTTTGGGGTCAGCG; MIP-2 forward 5′ACTGGTCCTGCTCCTCCTGCTG and reverse 5′TTGGTAGGGTCGTCAGGCATTG; KC forward 5′GCAGACAGTGGCAGGGATTC and reverse 5′GTGGCTATGACTTCGGTTTGG; and GAPDH forward 5′CAGCCTCGTCTCATAGACAAGATG and reverse 5′CAATGTCCACTTTGTCACAAGAGAA.

### 2.10. Statistical Analysis

Results are expressed as the mean ± standard error to the mean (SEM). Statistical differences were evaluated by one-way analysis of variance (ANOVA) followed by a Tukey multiple comparison* post hoc* test. All analyses were performed using GraphPad Prism 5.0 (GraphPad Software Inc). *p* value < 0.05 was considered significant.

## 3. Results

### 3.1. Effect of Inhaled Ammonium Vanadate on Lung Resistance

**Figure 1:**
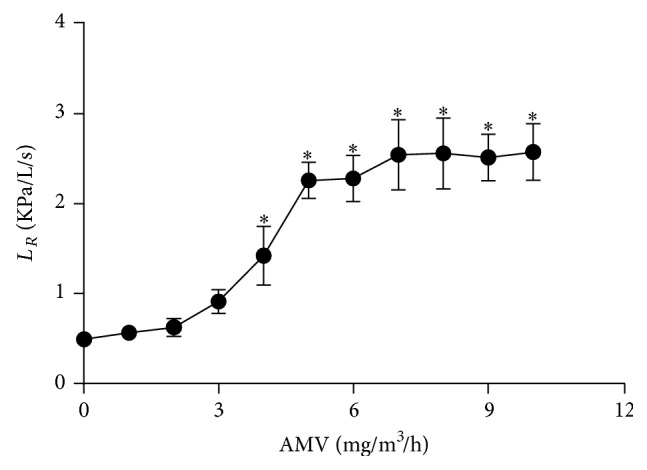
Dose-dependent lung resistance increases after inhalation of ammonium metavanadate aerosols (AMV). Rats received increasing doses of AMV aerosols for 15 minutes followed by 10 minutes of saline aerosols. Control animals received saline aerosols only. Lung resistance (*L*_*R*_) was determined as described in Materials and Methods. Values are expressed as means ± SEM (*n* = 8 for each experimental group). ^*∗*^ *p* < 0.05 based on one-way ANOVA followed by Tukey's multiple comparison* post hoc* test.

In order to determine the optimal dose of AMV required to induce airway hyperresponsiveness in our experimental model, rats were exposed growing concentrations of AMV aerosols while assessing lung resistances (*L*_*R*_) by pulmonary plethysmography. *L*_*R*_ increased proportionally with increased concentrations of AMV (Figure 1). *L*_*R*_ increased significantly in AMV-exposed rats from a dose of 4 mg/m^3^/h and reached a plateau at a dose of 5 mg/m^3^/h of AMV (2.26 ± 0.08 KPa/L/s, *p* < 0.05). Accordingly, we have used this latter dose of AMV in all subsequent experiments of the present study.

### 3.2. Inhaled PACAP Reversed the Airway Hyperresponsiveness Induced by AMV Exposure

**Figure 2:**
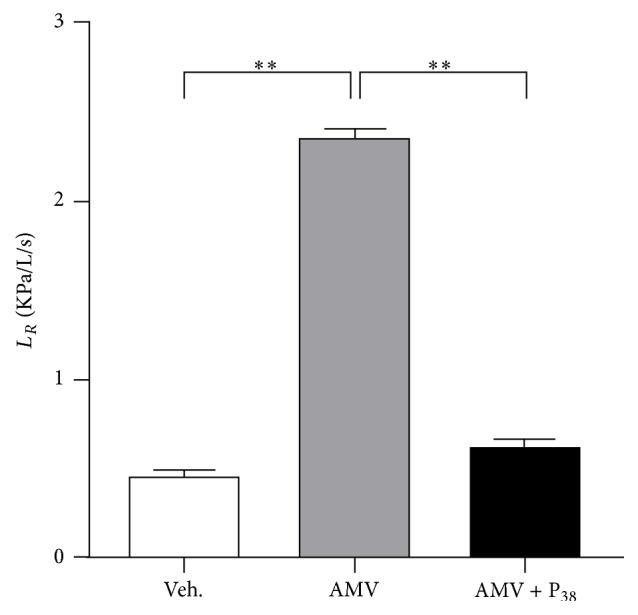
Inhalation of PACAP aerosols inhibits AMV-dependent increase of lung resistance. Rats received aerosols of AMV (5 mg/m^3^/h) for 15 minutes followed or not by PACAP aerosols (P_38_, 0.1 mM) for 10 minutes. Control rats inhaled vehicle (Veh.) only. Values are expressed as means ± SEM (*n* = 8 for each experimental group). ^*∗∗*^ *p* < 0.01 based on one-way ANOVA followed by Tukey's multiple comparison* post hoc* test.

As indicated by the dose response experiment, a significant increase of lung resistance was observed in rats exposed to vanadium for 15 minutes compared to animals treated with vehicle aerosols only (2.53 ± 0.05 versus 0.45 ± 0.02 KPa/L/s in control animals, *p* < 0.01; Figure 2). When AMV-sensitized rats were exposed to PACAP aerosols (0.1 mM) for 10 additional minutes, the increase of *L*_*R*_ was totally reversed to a level similar to that of the control animals (0.61 ± 0.03 KPa/L/s; Figure 2).

### 3.3. PACAP Inhalation Reduced the AMV-Induced Histological Alterations Observed in Lungs of Treated Rats

**Figure 3:**
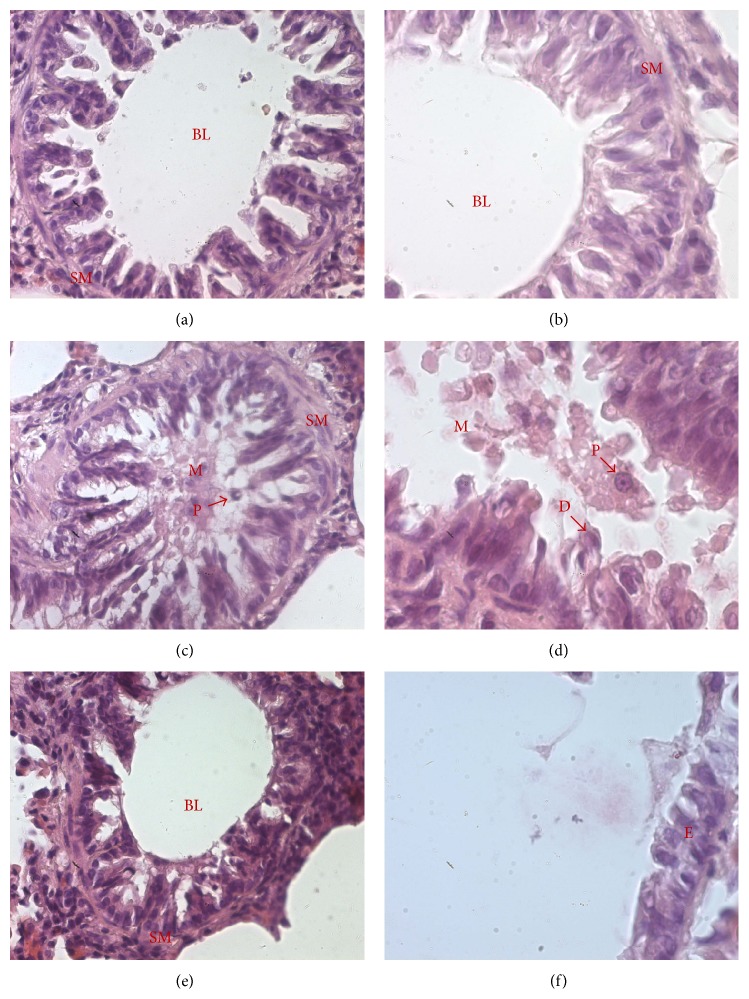
PACAP aerosols reverse the bronchoconstrictive effects of AMV exposure in rat lungs. Histological analysis of hematoxylin-eosin-stained lung sections from rats exposed to vehicle (a, b), AMV aerosols alone (c, d) or followed by inhalation of PACAP aerosols (e, f). Representative photomicrographs showing the structure of a representative bronchus at low (400x; a, c, and e) and higher (1000x; b, d, and f) magnification (*n* = 8 for each experimental group). BL: bronchial lumen; D: epithelium desquamation; E: bronchial epithelium; M: mucus; SM: smooth muscle layer; P: pyknotic nucleus.

**Figure 4:**
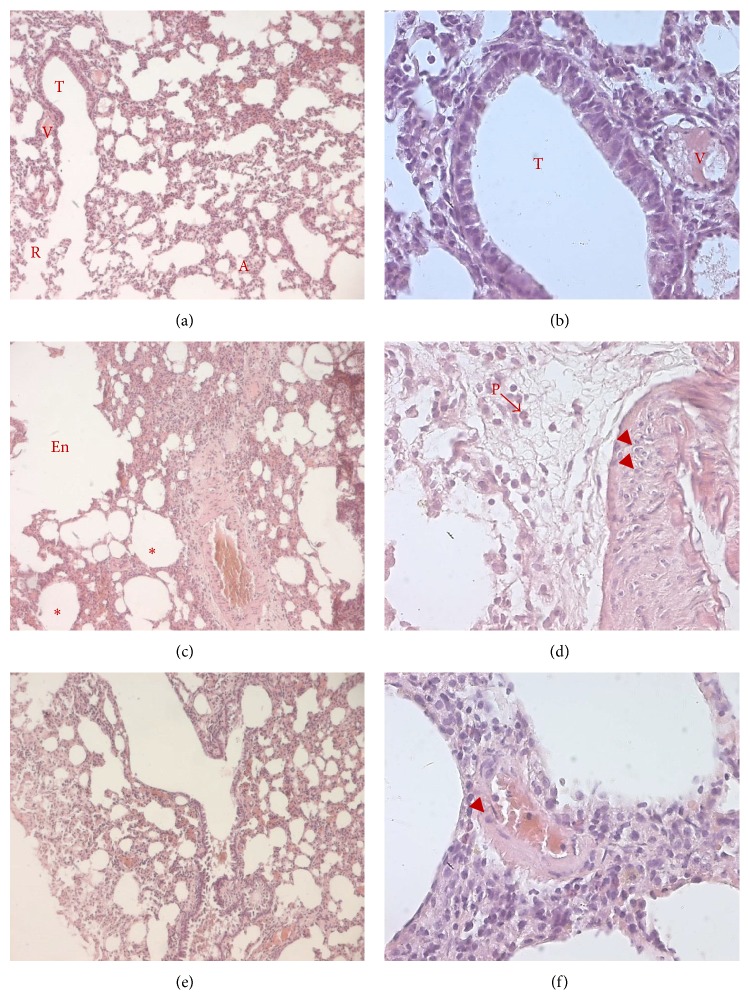
PACAP aerosols reduce AMV-induced inflammatory infiltration and colliquative necrosis in alveolar and bronchiolar tissues. Histological analysis of paraffin-embedded lungs stained with hematoxylin and eosin from animals exposed to vehicle (a, b), AMV aerosols alone (c, d) or followed by inhalation of PACAP aerosols (e, f). The presence of edema (asterisks), the enlargement of air spaces (En), and mononuclear cell infiltration (arrowheads) as well as the pyknotic nuclei (P) are indicated. Representative photomicrographs showing the structure of the alveolar and bronchiolar tissues at low (100x; a, c, and e) and higher (400x; b, d, and f) magnification (*n* = 8 for each experimental group). A: alveolus; En: air space enlargement; P: pyknotic nucleus; R: respiratory bronchiole; T: terminal bronchiole; V: pulmonary vessels.

As the AMV-dependent increase of lung resistance and its PACAP-mediated reversal may rely on structural changes of airways, we performed a histological study on lungs of treated rats. As expected lung sections of control rats stained with H&E showed normal bronchial lumen. Mucus was not abundant and smooth muscle (SM) layer had normal thickness (Figures 3(a) and 3(b)). In contrast, bronchial sections from rats exposed to vanadium alone showed various histological changes. AMV exposure induced an important accumulation of mucus leading to an obstructed aspect of bronchi. The obstruction appeared also enhanced by the observed hypertrophy of the smooth muscle layer (Figures 3(c) and 3(d)). These histological observations are further confirmed by morphometric analyses (Table 1) showing that the inhalation of vanadium decreased significantly the bronchial lumen diameter (BLD; 107.48 ± 4.77 versus 528.67 ± 9.19 *μ*m for control animals, *p* < 0.01) and increased significantly the airway smooth muscle layer thickness (ASMT; 39.52 ± 0.89 *μ*m versus 18.66 ± 0.35 *μ*m for control animals, *p* < 0.01) compared to vehicle-treated animals. Features of cell desquamation were also detected in AMV-treated rats (Figures 3(c) and 3(d)). Lung sections from rats that received PACAP aerosols following vanadium inhalation exhibited a less obstructed bronchial lumen with reduced mucus accumulation (Figures 3(e) and 3(f)). Moreover, the BLD and the airway smooth muscle layer thickness (ASMT) values were significantly decreased compared to vanadium-treated group and were comparable to those of the control group (479.09 ± 24.82 and 16.76 ± 0.41 versus 528.67 ± 9.19 and 18.66 ± 0.35 *μ*m, resp.; Table 1). Likewise, acute AMV exposure led to striking changes in alveolar tissue illustrated by the presence of edema, enlargement of air spaces, mononuclear cell infiltration, and colliquative necrosis characterized by numerous pyknotic nuclei (P) specifically in AMV-treated rats compared to control animals (Figures 4(a) and 4(d)). These AMV-associated histological alterations appeared strongly reduced in PACAP-treated animals (Figures 4(e) and 4(f)), suggesting a PACAP-counteracting action against the AMV-induced toxicity.

### 3.4. PACAP Treatment Counteracted the Prooxidative Response Consecutive to Lung AMV Exposure

**Figure 5:**
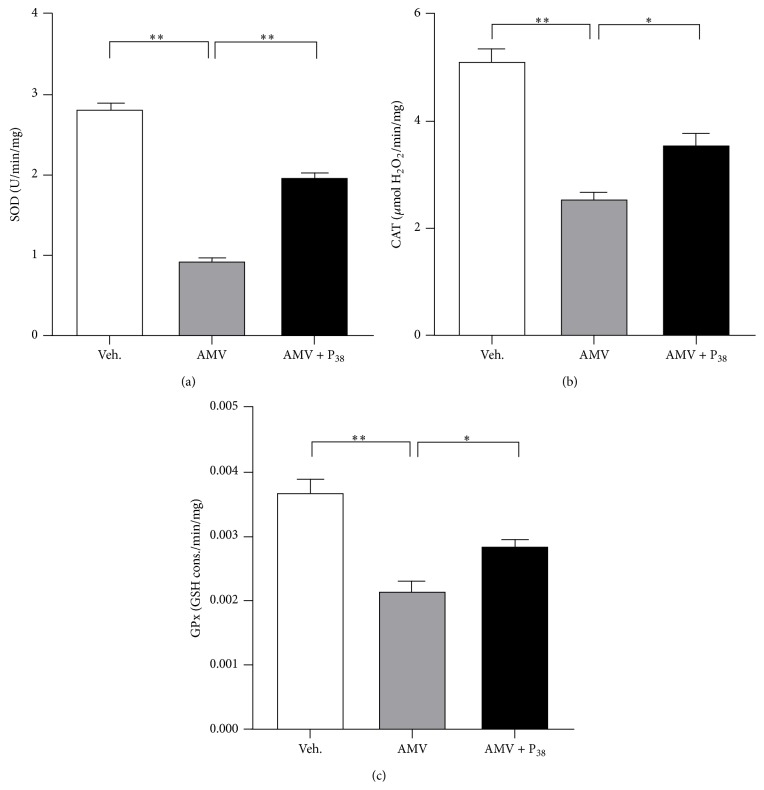
PACAP reverses the decrease of the activity of antioxidant enzymes (SOD, CAT, and GPx) in AMV-exposed rat lungs. The levels of SOD (a), CAT (b), and GPx (c) activities are determined on rat lung extracts from animals exposed to vehicle only (Veh.), AMV aerosols (AMV; 5 mg/m^3^/h) for 15 minutes followed or not by PACAP aerosols (AMV + P_38_; 0.1 mM) for 10 minutes. Values are expressed as means ± SEM (*n* = 8 for each experimental group). ^*∗*^ *p* < 0.05, ^*∗∗*^ *p* < 0.01 based on one-way ANOVA followed by Tukey's multiple comparison* post hoc* test.

The generation of reactive oxygen species (ROS) consecutive to AMV exposure could account, at least partly, for the vanadium-associated pulmonary toxicity. Then, we conducted analysis of the evolution of the oxidative status in lungs of animals exposed to vanadium and to vanadium followed by PACAP inhalation compared to control rats. Our results showed that neither AMV nor AMV + PACAP treatments have significant effects on lung malondialdehyde (MDA), NO, and H_2_O_2_ levels (Table 2). Nevertheless the lung reducing power, as assessed by the GSH/GSSG ratio, decreased by 60.8% specifically in the AMV-treated group compared to vehicle-exposed group (2.98 ± 0.16 versus 9.87 ± 0.34 resp.; Table 2). Similarly, the lung antioxidant activities of catalase (CAT), superoxide dismutase (SOD) and glutathione peroxidase (GPx) enzymes were significantly lower in AMV-treated rats (49.6%, 32.72%, and 58.3% than those of control group resp.; Figure 5). PACAP inhalation reversed significantly the AMV-dependent decrease of the lung reducing power (8.20 ± 0.37 for AMV + PACAP cotreated rats as compared to 2.98 ± 0.16 for AMV-exposed animals, *p* < 0.01; Table 2) and of the antioxidant enzyme activities (Figure 5). The AMV-dependent decrease of the SOD activity results in the significant inhibition of both Mn-SOD (0.36 ± 0.009 in AMV-treated group versus 0.97 ± 0.04 in control group, *p* < 0.01; Table 3) and Cu/Zn-SOD (0.08 ± 0.01 in AMV-treated group versus 0.29 ± 0.04 in control group, *p* < 0.01; Table 3) activities. Interestingly, PACAP treatment restored selectively and significantly the Mn-SOD activity (0.73 ± 0.01 in PACAP-treated group versus 0.36 ± 0.009 in AMV-treated group, *p* < 0.01; Table 3) but had no effect on the activity of the Cu/Zn-SOD isoform.

### 3.5. PACAP Modulated the Immune Cell Recruitment to Lungs Exposed to AMV Aerosols

As the oxidative status of the lungs could also reflect the activity of infiltrated innate immune cells, we evaluated in bronchoalveolar lavage fluid (BALF) the number of adherent and nonadherent cells recruited to the lungs. Following AMV inhalation, the number of nonadherent cells is significantly increased (5.39 ± 0.79 · 10^6^ cells in AMV-treated group compared to 2.74 ± 0.39 · 10^6^ cells in control group, *p* < 0.01; Table 4). In contrast, AMV exposure reduced significantly the number of alveolar macrophages (adherent cells) to almost half of basal levels (1.12 ± 0.16 · 10^6^ cells in AMV-exposed rats as opposed to 2.54 ± 0.19 · 10^6^ cells in control animals, *p* < 0.01). PACAP inhalation following AMV exposure reduced strongly the nonadherent cell number harvested from BALF (0.48 ± 0.13 · 10^6^ cells, *p* < 0.01) but had no consequence on the counted alveolar macrophages suggesting that the neuropeptide could act specifically on the recruitment of precise immune cell populations.

### 3.6. PACAP Inhibited the Vanadium-Induced Inflammatory Mediator Expression in Alveolar Macrophages

**Figure 6:**
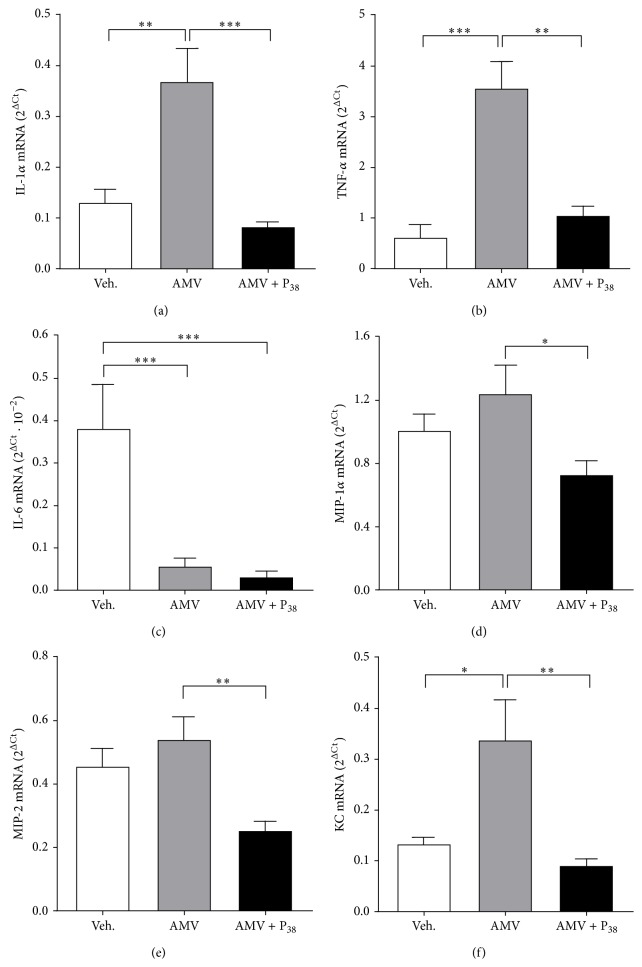
PACAP counteracts the proinflammatory properties of AMV on bronchoalveolar macrophages. The levels of expression of Il-1*α* (a), TNF-*α* (b), Il-6 (c), MIP-1*α* (d), MIP-2 (e), and KC (f) are determined by quantitative PCR on mRNAs extracted from alveolar macrophages. The macrophages are isolated by bronchoalveolar lavages 4 hours after treatment of rats with vehicle aerosols only (Veh.), AMV aerosols (AMV; 5 mg/m^3^/h) followed or not by PACAP aerosols (AMV + P_38_; 0.1 mM). Values are expressed as means ± SEM of the 2^ΔCt^ values (*n* = 8 for each experimental group). ^*∗*^ *p* < 0.05, ^*∗∗*^ *p* < 0.01, ^*∗∗∗*^ *p* < 0.001 based on one-way ANOVA followed by Tukey's multiple comparison* post hoc* test.

To further study the AMV-dependent increase of the local inflammatory response and the potential counteracting actions of inhaled PACAP, we determined by real time quantitative PCR the expression levels of mRNA encoding the proinflammatory cytokines IL-1*α*, TNF-*α*, and IL-6 and the chemokines macrophage inflammatory protein-1*α* (MIP-1*α*), macrophage inflammatory protein-2 (MIP-2), and keratinocyte chemoattractant (KC) in alveolar macrophages isolated from BALF of treated animals. Vanadium exposure increased significantly the expression levels of Il-1*α*, Tnf-*α*, and KC mRNA (Figures 6(a), 6(b), and 6(f)) illustrating the proinflammatory consequences of AMV inhalation and decreased strongly the expression level of Il-6 mRNA (Figure 6(c)). The PACAP inhalation reduced significantly the AMV-dependent upregulation of Il-1*α* (*p* < 0.001), TNf-*α* (*p* < 0.01), and KC (*p* < 0.01) expression but had no effect on the AMV-induced downregulation of the Il-6 gene expression (Figure 6). Furthermore, PACAP downregulated significantly both MIP-1*α* (*p* < 0.05) and MIP-2 (*p* < 0.01) gene expression.

### 3.7. PACAP Had No Effect on Lung Vanadium Content

As shown in Table 5, no detectable vanadium was present in control lungs. PACAP had no significant effect on vanadium accumulation in lung tissue (2.38 ± 1.14 versus 2.32 ± 0.91 *μ*g/g of wet tissue in AMV-treated group, *p* > 0.05).

## 4. Discussion

The present study investigated the toxic effects of ammonium vanadate in rat lung and the therapeutic potential of the neuropeptide PACAP.

According to the Agency for Toxic Substances and Disease Registry's report, in vanadium, the toxic compounds are vanadium pentoxide (V_2_O_5_), sodium metavanadate (NaVO_3_), sodium orthovanadate (Na_3_VO_4_), vanadyl sulfate (VOSO_4_), and ammonium metavanadate (NH_4_VO_3_) [31]. Very few studies have compared the differential toxicity of vanadium compounds. However, their toxicity appears tightly associated with their physicochemical state [32]. In fact, vanadium compounds present six oxidation states with 3+, 4+, and 5+ states being the most common [33]. In living systems, the vanadium exists under two main forms, the anionic vanadate and the cationic vanadyl forms which could give rise to each other through a reduction/oxidation process. These multivalent forms present a differential toxicity closely related to their valence [1, 34]. Thereby the pentavalent vanadate^+5^ presents a higher immunotoxicity in lungs compared to the tetravalent vanadyl^+4^ or the trivalent[V(III)] bis(dipicolinato)vanadium^+3^, showing that the oxidation state of vanadium could affect its toxicity [34]. Additionally, the solubility of the vanadium compounds is another critical characteristic affecting their toxicity both after inhalation [34, 35] and enteral administration [36]. Indeed, as illustrated by the work of Cohen et al., the pentavalent insoluble vanadium pentoxide (V_2_O_5_) presents a reduced pulmonary toxicity compared to the pentavalent soluble sodium metavanadate and ammonium metavanadate [34, 35]. This solubility dependent toxicity has been proposed to be associated in part with the clearance and related processes of the compounds from the tissue. Thus, if 90% of an instilled dose of NaVO_3_ was cleared from exposed lungs within 24 h, only 40% of a similar dose of NH_4_VO_3_ administered by aerosol inhalation was cleared during the same period [35, 37]. This differential clearance rate from the lungs, which correlates with the differential solubility of the two compounds, has been proposed to support the increased toxicity of NH_4_VO_3_ compared to an equivalent dose of NaVO_3_ after inhalation.

Based on the data exposed above, we used the most toxic form of vanadium (pentavalent NH_4_VO_3_ compound) for its high solubility at neutral pH, its significant concentration in dusts and fumes, and its latency to form oligomers.

In our experiments, lung toxic effects of vanadium poisoning were found (bronchoconstriction, mucus secretion, recruitment of immune cells, and oxidative stress) after a single exposure to the component. This could be interesting when compared to other studies which used repeated exposure protocols with a longer time of exposure to generate the same effects [35, 38–40]. One of the objectives of the present work was the development of a rat model of acute pulmonary toxicity after a single exposure of aerosol particles of ammonium vanadate (AMV). That is why we tested the effect of increasing doses of AMV. Then we choose the lower efficient dose (5 mg/m^3^) that elevated *L*_*R*_ and did not exceed the LC 50 of AMV after inhalation in rats which is 7.8 mg/m^3^ for 4 hours [31].

We did not include an additional control group of animals receiving an equivalent dose of ammonium ion to study any potential variations due to the cation. Nevertheless, previous studies have demonstrated in a murine model of intraperitoneal administration of ammonium metavanadate that the ammonium ion does not interfere with the vanadium toxicity on the immune compartment [41, 42]. Moreover, this experimental design is similar to that published by Cohen et al. in 1996 based on rats repeatedly exposed to ammonium metavanadate aerosols [35]. In this model of vanadium-dependent pulmonary toxicity, the authors, based on previous studies mentioned above, considered that the observed effects resulted from the vanadium toxicity only and not from ammonium pulmonary concentration. In addition, as the vanadium burden in the lungs of exposed rats in our model of airway hyperresponsiveness is inferior to that measured in Cohen's study after 4 days of exposure to ammonium metavanadate aerosols (2.32 ± 0.91 *μ*g/g of wet tissue versus 26.9 *μ*g/g of wet tissue) (Table 5), we can estimate that in our model the achieved pulmonary ammonium concentration should be far less than those obtained in Cohen's work. In consequence, we could reasonably assume that the observed effects reported in our work do not rely on potential actions of the supplied ammonium ion. The cations (ammonium or sodium) do not seem to interfere with the molecular mechanisms responsible for the toxicity of the vanadium compounds whose toxicity seems to be supported by the action of the vanadate and vanadyl forms of the molecule on different enzymatic systems [43–47]. On the other hand, we used a control group that received NaCl aerosols that did not change the basal control values.

In our case, this vanadium pulmonary accumulation caused an increase of total lung resistance consecutive to the contraction of the airway smooth muscle layer and mucus secretion in narrowing bronchial lumen (Figures 2 and 3, Table 1). It has been suggested from clinical and animal studies that vanadate acts directly on the smooth muscle of airways, promoting the release of intracellular calcium from an intracellular store [4]. The vanadate-induced spasm is not mediated by the release of parasympathetic or sympathetic neurotransmitters. Vanadate appears to enter the cytoplasm of smooth muscle cells via anion exchange pathways to inhibit calcium-ATPase system, thereby increasing the cytosolic calcium (Ca^2+^) and causing contraction. The process of vanadate-induced Ca^2+^ release involves both the production of inositol phosphate second messengers and inhibition of Ca-ATPase. The activation of PKC plays also an important role in this contraction [3, 4].

Previous studies demonstrate that PACAP-containing nerve fibres are found in association with bronchial smooth muscle in primates and rodents [48] and a moderate number of PACAP-like immunoreactive nerve fibers were seen in association with human bronchial and vascular smooth muscles and around seromucus glands, suggesting that PACAP-38 may play a role in the endogenous regulation of airway tone [11]. PACAP-38 represents up to 80–90% of the total PACAP in the body and has longer lasting bronchodilator activity on constricted airways than its shorter fragment, PACAP-27,* in vitro* as well* in vivo*. Inhaled PACAP-38 also causes more sustained inhibition of bronchoconstriction* in vivo* than VIP, without cardiovascular side effects at doses inhibiting bronchoconstriction [10, 49, 50]. Our study demonstrates that PACAP inhalation had a bronchodilator effect in rats, previously inhaled with AMV (Figure 2, Table 1). The mechanism by which PACAP induces bronchodilatation against AMV-induced bronchoconstriction is not fully understood yet and needs further investigations.

Vanadium is known to avidly permeate cell membranes. Once inside the cells, vanadium binds to many intracellular ligands and is presumably detoxified [51]. When vanadium load exceeds the capacity of chelators, vanadyl (V^4+^) and the more potent vanadate (V^+5^) ions may be released at high local concentration and may target important biomolecules resulting in toxic effects [52]. Vanadate accumulation in lungs induced an oxidative stress characterized by decreased reducing power (GSH/GSSG ratio) (Table 2) and inhibition of antioxidant enzyme activities such as CAT, GPx, and SOD (Figure 5). The decreased activity of the antioxidant system associated to vanadium toxicity is in agreement with previous published data [53, 54]. A reduced activity of SOD and CAT may lower their cellular efficiency to detoxify these potentially active oxyradicals [55]. GSH is the major nonenzymatic antioxidant of the cell and its depletion may explain the features of necrosis observed in our study (Table 2, Figure 4(d)). Similarly, it was found that intratracheal instillation of vanadium resulted in histopathological changes, such as desquamation and degeneration of swollen bronchiolar epithelium, hyperplasia of goblet cells, diffuse haemorrhage, effusions of fibrin, and pulmonary edema [56]. In the presence of NADPH, several flavoenzymes are able to reduce vanadate (V) to generate vanadium (IV). During the reduction process, molecular oxygen is consumed to generate ^∙^O_2_^−^ which undergoes a dismutation to H_2_O_2_ in the presence of superoxide dismutase (SOD). Vanadium (IV) is able to generate ^∙^OH radical from H_2_O_2_ via a fenton-like reaction [5, 57]. This H_2_O_2_ consumption could be linked to the absence of H_2_O_2_ level rise in lungs of rats challenged with vanadate (Table 2). ^∙^OH is one of the most powerful oxidants and can attack polyunsaturated fatty acids in membranes (lipid peroxidation) and many other biological molecules [53, 58]. It has been proposed that the resulting reactive species generated by vanadate from H_2_O_2_ and lipid hydroperoxide* via* a fenton-like reaction may play a significant role in the mechanism of vanadate-induced cellular injury [59]. The vanadate action on lipid peroxidation is tightly associated with experimental models, tissues, and species in which the process is evaluated. In a model of rat alloxan-induced diabetes, the administration of sodium orthovanadate normalized the levels of antioxidant enzymes and reduced lipid peroxidation in brain [60]. In contrast in a murine model of high fat diet, mice receiving orally administered ammonium metavanadate present more severe physiopathological changes compared to normal diet fed animals. In this context, Imura et al. [36] reported that NH_4_VO_3_ administration induced small intestine tissue necrosis through lipid accumulation, increased oxidative stress, and lipid peroxidation. Similarly, Ścibior et al. [61] reported that 18-week sodium metavanadate intoxication enhanced spontaneous MDA generation in rat liver. In our model, acute AMV exposure did not affect significantly lung lipid peroxidation and H_2_O_2_ levels (Table 2) despite decreased activities of SOD, CAT. and GPx antioxidant enzymes (Figure 5). These observations are corroborated by no significant differences in production in lung homogenates from exposed and nonexposed animals (Table 2). Then our observations are in line with previous data reporting no variation of lipoperoxidationin in different tissues after ammonium metavanadate inhalation or oral administration of vanadium sulfate [35, 62, 63].

Moreover, our results showed that lung toxicity does not occur through a carbon monoxide (CO) mediated mechanism and PACAP had no effects on NO lung levels (Table 2). However, the relaxant effect of PACAP was shown to be mediated via an NO/cGMP-dependent and NO-dependent transduction pathways [64]. There are no data about NO-mediated lung tissue toxicity after vanadate inhalation in rodents and humans and such mechanism is still unknown. Available data concerned the effects of several vanadium compounds on macrophages and endothelial cells. In fact, vanadate inactivates protein tyrosine phosphatase and enhances tyrosine phosphorylation [65].* In vitro*, sodium orthovanadate at 500 *μ*M alone caused a mild but significant increase in NO production of J774A.1 mouse macrophage cells by 24 hours of incubation [66]. Similar NO induction was recorded after exposure of osteoblast-like cells to vanadate at concentrations over 50 *μ*M [67]. Moreover, vanadate caused endothelium-dependent relaxations in isolated porcine coronary arteries by synthesis of endothelium-derived NO [68]. In contrast, VO_4_ increased tyrosine phosphorylation of the nitric oxide synthase (NOS) which reduced both NOS activity and NO bioavailability [69]. These contradictory effects may be related to the presence of many complex mechanisms of NO induction which seem to be dose- and tissue-specific and also depend on *t* exposure duration to the metal and its redox state.

Treatment of the vanadate-inhaling animals with PACAP restored the reducing power and the activities of the antioxidant enzymes (Figure 5). Thus we suggest that the improvements of lung injury in vanadate exposed animals could be mediated by an antioxidant mechanism. In this report, PACAP increased significantly Mn-SOD activity, but not the activities of the extracellular and cytosolic isoforms (Table 3) suggesting that PACAP has a mitochondrial site of accumulation and/or a selective affinity to this isoform.

In addition to its bronchoconstrictor effects, ammonium metavanadate can induce pulmonary inflammation as indicated by the significant increase of IL-1*α* and TNF-*α* expression levels in the AMV-treated rats (Figure 6). Surprisingly, the number of alveolar macrophages appears lower in AMV-, compared to vehicle-treated rats (Table 4). The increased expression of alveolar macrophage-derived proinflammatory factors in association with the inflammatory environment observed in histological analyses reveals undoubtedly activation of alveolar macrophages in AMV-exposed rats. Hence, the reduced number of alveolar macrophages in AMV-treated BALF may rely more probably on an increased adhesion of macrophages to the tissue (making them resistant to bronchoalveolar lavages) rather than an AMV-dependent reduction of macrophage recruitment. Furthermore, the histopathological features observed on lung sections reveal an important infiltration of polymorphonuclear leukocytes (PMNs) (Figures 4(c) and 4(d)) corroborated by increased numbers of nonadherent cells in BALF of AMV-exposed rats (Table 4). These results are consistent with other reports indicating that sodium metavanadate (NaVO_3_) produced a 20–40% increase in PMNs during peak inflammation observed four hours after NaVO_3_-instillation in rat lungs. A significant neutrophil influx was also detected following the instillation of vanadyl sulfate (VOSO_4_) and vanadium pentoxide (V_2_O_5_) [6, 70].

The AMV-induced PMN influx into the pulmonary parenchyma could be driven by the significantly increased expression levels of the proinflammatory chemokine KC (Figure 6(f)) in isolated alveolar macrophages which is known as a potent neutrophil chemotactic/activating factor [6]. In contrast to an* in vitro* study reporting that exposure of murine macrophage cell line Raw 264.7 to NaVO_3_ *in vitro* induced MIP-2 mRNA expression [70], the expression levels of both MIP-1*α* (Figure 6(d)) and MIP-2 (Figure 6(e)) are not significantly regulated following AMV exposure* in vivo*. The fact that the expression levels of these chemokines remained relatively elevated in vanadate-exposed rats compared to vehicle-treated animals suggests that the vanadate exposure could stimulate the expression of MIP-1*α* and MIP-2 in alveolar macrophages but in a different time window than those we assessed.

Our results showed that PACAP inhalation following AMV exposure* in vivo* powerfully decreased the AMV-dependent upregulation of the expression of the proinflammatory cytokines IL-1*α* (Figure 6(a)) and TNF-*α* (Figure 6(b)), as well as those of the chemokines MIP-1*α* (Figure 6(d)), MIP-2 (Figure 6(e)), and KC (Figure 6(f)), which results in a substantial reduction in the PMNs influx (Figures 4(e) and 4(f)), in the number of alveolar macrophages (Table 4) and in mucus production (Figures 3(e) and 3(f)). Our observations are in line with previous work showing that PACAP inhibits MIP-1*α*, MIP-2, and KC expression in LPS-stimulated murine peritoneal macrophages and Raw 264.7 cell line [71] and reduces chemotaxis of nonactivated and N-formyl-L-methionyl-L-leucyl-L-phenylalanine stimulated neutrophils [72]. The reduction of the alveolar macrophage number induced by PACAP administration could result from a combined action of two processes. Firstly, PACAP stimulates the adherence capacity of macrophages through protein kinase C (PKC) activation [73], making the cells more resistant to BAL similarly to what is observed for AMV-activated macrophages. Secondly, reduction of BALF alveolar macrophages in PACAP-treated rats (Table 4) could be supported by the PACAP-dependent downregulation of MIP-1*α* and MIP-2 mRNA expression observed in our results (Figures 6(d) and 6(e), resp.).

Supplementation with PACAP improved vanadate-induced lung adverse effects without significantly reducing metal accumulation in the tissue (Table 5).

## 5. Conclusion

Our data highlight the link between vanadium accumulation into lung tissue, oxidative stress and pulmonary dysfunction, and the therapeutic potential of the neuropeptide PACAP to counteract the vanadate-induced toxicity. This is the first study to our knowledge showing that PACAP-38 has powerful bronchodilator, anti-inflammatory, and antioxidant effects in rats treated with AMV. The neuropeptide PACAP could restore the ventilatory function in AMV-exposed animals directly by potential actions on smooth muscle cells decreasing their contractile function, on goblet cells decreasing their mucus production and indirectly by inhibiting the local inflammatory response and its associated processes such as oxidative stress and immune cell infiltration. This experimental study showed that supplementation of PACAP by aerosol inhalation was safe and exerts potent protective action on lung exposed to vanadate suggesting that PACAP administration by the nasal route could be a promising therapy for treatment of lung inflammatory diseases. Nevertheless, further studies are needed to clearly establish the mechanistic basis of AMV toxicity and PACAP-dependent protection with consideration given to biochemical factors. In this context, the signaling pathways leading to the activation of the transcription factor NF-*κ*B which are known to be differently regulated by PACAP and vanadium should be explored.

## Figures and Tables

**Table 1:** Effects of PACAP on lung histomorphological parameters, airway smooth muscle layer thickness (ASMT), and bronchial lumen diameter (BLD) in AMV-induced lung toxicity. Rats received AMV aerosols (AMV; 5 mg/m^3^/h) for 15 minutes followed or not by PACAP aerosols (AMV + P_38_; 0.1 mM) for 10 additional minutes. Control rats (controls) received the vehicle only. Values are expressed as means ± SEM (*n* = 8 for each experimental group). ^*∗∗*^ *p* < 0.01 versus controls; ^##^ *p* < 0.01 versus AMV in Tukey's multiple comparison *post  hoc* test.

	ASMT (*µ*m)	BLD (*µ*m)
Controls	18.66 ± 0.35	528.67 ± 9.19
AMV	39.52 ± 0.89^*∗∗*^	107.48 ± 4.77^*∗∗*^
AMV + P_38_	16.76 ± 0.41^##^	479.09 ± 24.82^##^

**Table 2:** Effects of PACAP on lung oxidative status in AMV-induced lung toxicity. Rats received AMV aerosols (AMV; 5 mg/m^3^/h) for 15 minutes followed or not by PACAP aerosols (AMV + P_38_; 0.1 mM) for 10 additional minutes. Control rats (controls) received the vehicle only. Values are means ± SEM (*n* = 8 for each experimental group). ^*∗∗*^ *p* < 0.01 versus control; ^#^ *p* < 0.05 versus AMV; ^##^ *p* < 0.01 versus AMV in Tukey's multiple comparison *post  hoc* test.

	Controls	AMV	AMV + P_38_
GSH (nmol/mg)	57.97 ± 1.95	26.02 ± 1.29^*∗∗*^	47.99 ± 2.84^##^
GSSG (nmol/mg)	5.91 ± 0.19	8.78 ± 0.17^*∗∗*^	6.01 ± 0.43^#^
GSH/GSSG	9.87 ± 0.34	2.98 ± 0.16^*∗∗*^	8.20 ± 0.37^##^
MDA (nmol/mg)	0.15 ± 0.03	0.17 ± 0.02	0.16 ± 0.01
H_2_O_2_ (mmol/mg)	24.76 ± 1.80	26.43 ± 2.38	28.36 ± 2.61
NO metabolites (*µ*mol/mg)	24.70 ± 1.08	29.99 ± 3.75	31.37 ± 2.62

**Table 3:** PACAP's regulatory effects on the enzymatic activity of the Mn-SOD and Cu/Zn-SOD isoforms in AMV-induced lung toxicity. Rats received AMV aerosols (AMV; 5 mg/m^3^/h) for 15 minutes followed or not by PACAP aerosols (AMV + P_38_; 0.1 mM) for 10 additional minutes. Control rats (controls) received the vehicle only. Values are expressed as means ± SEM (*n* = 8 for each experimental group). ^*∗*^ *p* < 0.05 versus control; ^*∗∗*^ *p* < 0.01 versus control; ^##^ *p* < 0.01 versus AMV in Tukey's multiple comparison *post  hoc* test.

	Mn-SOD (SOD units/min/mg)	Cu/Zn-SOD (SOD units/min/mg)
Controls	0.97 ± 0.04	0.29 ± 0.04
AMV	0.36 ± 0.009^*∗∗*^	0.08 ± 0.01^*∗∗*^
AMV + P_38_	0.73 ± 0.01^##,*∗*^	0.08 ± 0.01^*∗∗*^

**Table 4:** Effect of PACAP aerosols on adherent cell and nonadherent cell numbers in bronchoalveolar lavage fluids (BALF) of AMV-exposed rats. Rats received AMV aerosols (AMV; 5 mg/m^3^/h) for 15 minutes followed or not by PACAP aerosols (AMV + P_38_; 0.1 mM) for 10 minutes. Control rats (controls) received the vehicle only. Four hours after treatment, the treated rats were submitted to bronchoalveolar lavages. The numbers of adherent and nonadherent cells contained in BALF were further determined after 24 h of culture. Values are expressed as means ± SEM (*n* = 8 for each experimental group). ^*∗∗*^ *p* < 0.01 versus control; ^##^ *p* < 0.01 versus AMV in Tukey's multiple comparison *post  hoc* test.

	Adherent cells (×10^6^)	Nonadherent cells (×10^6^)
Controls	2.54 ± 0.19	2.74 ± 0.39
AMV	1.12 ± 0.16^*∗∗*^	5.39 ± 0.79^*∗∗*^
AMV + P_38_	0.64 ± 0.14^##,*∗∗*^	0.48 ± 0.13^##,*∗∗*^

**Table 5:** Lung vanadium contents after AMV and/or PACAP treatments. Rats received AMV aerosols (AMV; 5 mg/m^3^/h) for 15 minutes followed or not by PACAP aerosols (AMV + P_38_; 0.1 mM) for 10 additional minutes. Control rats (controls) received the vehicle only. Values are expressed as means ± SEM (*n* = 8 for each experimental group). ^*∗∗*^ *p* < 0.01 versus controls (Tukey's multiple comparison *post  hoc* test).

	Controls	AMV	AMV + P_38_
AMV (*µ*g/g)	UQL	2.32 ± 0.91^*∗∗*^	2.38 ± 1.14^*∗∗*^

UQL: under the quantification limit.
